# A mHealth cardiac rehabilitation exercise intervention: findings from content development studies

**DOI:** 10.1186/1471-2261-12-36

**Published:** 2012-05-30

**Authors:** Leila Pfaeffli, Ralph Maddison, Robyn Whittaker, Ralph Stewart, Andrew Kerr, Yannan Jiang, Geoff Kira, Karen Carter, Lance Dalleck

**Affiliations:** 1 Clinical Trials Research Unit, University of Auckland, Auckland, New Zealand; 2 Department of Medicine, University of Auckland, Auckland, New Zealand; 3 Department of Epidemiology and Biostatistics, University of Auckland, Auckland, New Zealand; 4 Department of Sport and Exercise Science, University of Auckland, Auckland, New Zealand

**Keywords:** Cardiac rehabilitation, Exercise, Telemedicine, Internet

## Abstract

**Background:**

Involving stakeholders and consumers throughout the content and study design ensures interventions are engaging and relevant for end-users. The aim of this paper is to present the content development process for a mHealth (mobile phone and internet-based) cardiac rehabilitation (CR) exercise intervention.

**Methods:**

An innovative mHealth intervention was developed with patient input using the following steps: conceptualization, formative research, pre-testing, and pilot testing. Conceptualization, including theoretical and technical aspects, was undertaken by experts. For the formative component, focus groups and interviews with cardiac patients were conducted to discuss their perceptions of a mHealth CR program. A general inductive thematic approach identified common themes. A preliminary library of text and video messages were then developed. Participants were recruited from CR education sessions to pre-test and provide feedback on the content using an online survey. Common responses were extracted and compiled. An iterative process was used to refine content prior to pilot testing and conduct of a randomized controlled trial.

**Results:**

38 CR patients and 3 CR nurses participated in the formative research and 20 CR patients participated in the content pre-testing. Participants perceived the mHealth program as an effective approach to inform and motivate patients to exercise. For the qualitative study, 100% (n = 41) of participants thought it to be a good idea, and 11% of participants felt it might not be useful for them, but would be for others. Of the 20 participants who completed the online survey, 17 out of 20 (85%) stated they would sign up to a program where they could receive information by video messages on a website, and 12 out of 20 (60%) showed interest in a texting program. Some older CR patients viewed technology as a potential barrier as they were unfamiliar with text messaging or did not have mobile phones. Steps to instruct participants to receive texts and view the website were written into the study protocol. Suggestions to improve videos and wording of texts were fed back to the content development team and refined.

**Conclusions:**

Most participants thought a mHealth exercise program was an effective way to deliver exercise-based CR. The results were used to develop an innovative multimedia exercise intervention. A randomized controlled trial is currently underway.

**Trial registration:**

ACTRN12611000117910

## Background

Cardiac rehabilitation (CR) is an integral part of the management of people with cardiovascular disease (CVD) and is a cost-effective way to improve patients’ physical and psychological health [1,2]. CR can involve a variety of components, including medical evaluation, prescribed exercise, and education to reduce cardiac risk factors [3]. Exercise is an essential component of CR. Exercise-based CR programs have demonstrated reductions in morbidity and mortality in patients with CVD [3] while increasing patients’ exercise capacity and fitness [1]. The benefits of CR are well-known yet provision is inadequate in all countries in which it has been measured [4]. In the United Kingdom, the majority of patients are not invited to take part in CR or choose not partake [1]. Low uptake rates have also been reported in other developed countries, including the U.S. [5], Canada [6], Australia [7], and New Zealand [8,9].

Common reasons patients cannot or choose not to attend CR include lack of access to transport, issues around parking, ill health, or domestic responsibilities [10]. Younger patients are less likely to attend, due to time or scheduling commitments associated with returning to work [8].

Current delivery approaches do not suit all people [11,12] and innovative approaches are needed to improve the delivery and utilization of CR. A range of different options for rehabilitation delivery should be available for people according to their preferences and needs [13]. Telephone-based interventions can be effective in supporting behavior change [14,15], however, the use of mobile phone and Internet-based technologies for program delivery (‘mHealth’) may offer an effective and efficient way to reach more people [16].

Two recent (2009, 2010) systematic reviews support the delivery of short-message service (SMS) [17] and internet [18] interventions for achieving behavior change. For SMS, 13 of the 14 studies reviewed resulted in positive behavior change outcomes for smoking cessation and diabetes management. These types of mHealth programs can be delivered anywhere at any time, facilitating regular communication. Messages can be delivered in a time-sensitive or time-appropriate manner, and participants are not required to attend a clinic. This makes programs far more proactive (initiated by the service) than traditional services, which often require action by the participant before they impart information or provide support. The flexible nature of the programs means they can be tailored to specific cultural, sex and age groups.

MHealth programs have potential to improve access and delivery of CR, particularly among those unable or unwilling to attend center-based CR. A randomized controlled trial (RCT) to determine the effect of a mHealth exercise-based CR program on exercise capacity is currently underway [19]. The intervention included exercise prescription and behavior change strategies delivered by SMS and brief video vignettes via a secure participant website. The website contained additional interactive features, such as goal setting charts and graphs to record time spent being physically active. This paper outlines the content development process of the mHealth exercise-based CR intervention and includes the results from formative and pre-testing studies.

## Methods and results

**Figure 1:**
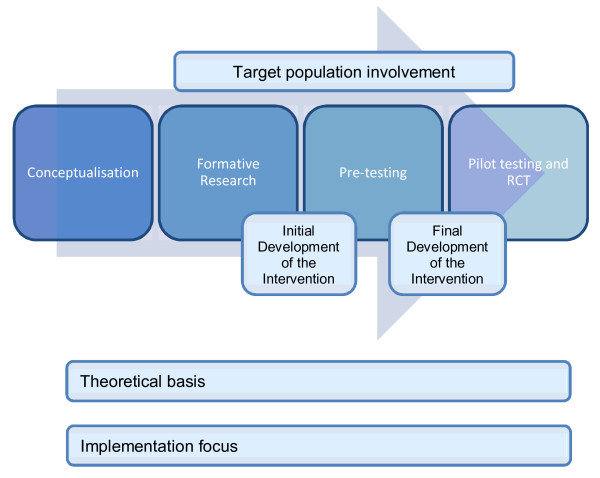
**Content development process steps **[20].

The intervention content development followed a series of steps used in previous mHealth interventions: conceptualization, formative research to inform the development, pre-testing content, pilot study, pragmatic randomized controlled trial, and further qualitative research [20]. This process is summarized in Figure 1 and discussed in detail below. The methods and results are presented for each step. Ethics approval for a qualitative study (NTX/10/02/006) and an online survey (NTX/10/10/099) were obtained from the Northern X Regional Ethics Committee.

### Conceptualization

An expert content advisory group was developed consisting of two cardiologists, a cardiac rehabilitation nurse specialist (CRN), two exercise scientists, a behavioral researcher, an expert in mobile phone delivered interventions [21,22] and a M?ori (Indigenous) health researcher. The group met weekly during the 6 month planning stage to determine the overall direction of the intervention, including the content to be included and how it would be delivered. Appropriate behavior change theories [23] were considered, as well as how to adapt existing American College of Sports Medicine (ACSM) guidelines for exercise-based CR [24] and behavior change strategies into SMS (160 character text messages) and video format. As a result, important themes for exercise prescription content, behavior change theory and strategies, the purpose of role modeling, and the technology platform to deliver this content were identified prior to formative research.

### Study 1: formative research methods

Focus groups and individual telephone interviews were conducted with people with CVD who had attended (attenders), and those who were invited but never attended (non-attenders), a CR program. During this formative stage, we evaluated participants’ beliefs and perceptions of the CR process, perceived barriers, and perceptions of the proposed mHealth CR program of SMS, video messages, and an interactive website.

Attenders were recruited from existing center-based CR programs through established relationships with hospital staff (CRNs). The corresponding author attended the sessions and recruited participants directly. All interested attenders were invited to take part in a focus group, which were conducted the following week after the CR session. In a few cases attenders wanted to take part but could not attend the focus group. These participants were then interviewed over the telephone. In total, 4 focus groups and 5 telephone interviews were conducted in various regions across one city to recruit a more diverse sample.

Non-attenders were recruited by CRNs, who retrospectively reviewed patient records to identify people that had been invited, but did not attend CR. For this group, we conducted one-on-one semi-structured telephone interviews. A key informant focus group was also conducted with three CRNs, who were involved in the recruitment process for this study. Interviews and focus groups were audiotaped and transcribed, and were conducted by the corresponding author.

A general inductive thematic approach was used to identify common themes and meanings from the data [25]. NVivo9© was used to group items together based on the codes and comments in the margins. Once all data were collected, categories and sub-categories were organized and refined into themes created during analysis. Data were coded and analyzed by the corresponding author and then discussed with the content advisory group.

### Formative research results: focus groups and telephone interviews

In total, 41 participants took part in the qualitative research, comprised of 28 attenders, 10 non-attenders, and three CRNs. Demographic characteristics of attenders and non-attenders are summarized in Table 1.

**Table 1:** Demographics for qualitative study (N = 38) and pre-testing study (N = 20)

**Demographics**	**Formative research**			**Pre-testing study**
	**Attenders**	**Non-Attenders**	**Overall**	**(n = 20)**
	**(n=28)**	**(n=10)**	**(n=38)**	
Gender				
Male	19 (68%)	5 (50%)	24 (63%)	14 (70%)
Female	9 (32%)	5 (50%)	14 (37%)	6 (30%)
Age (in years)				
≤35	2 (7%)	1 (10%)	3 (8%)	0
36–45	4 (14%)	1 (10%)	5 (13%)	Z2 (10%)
46–55	2 (7%)	2 (20%)	4 (11%)	4 (20%)
56–65	6 (21%)	2 (20%)	8 (21%)	7 (35%)
66–75	11 (39%)	1 (10%)	12 (32%)	6 (30%)
≥76	3 (11%)	3 (30%)	6 (16%)	1 (5%)
Ethnicity				
New Zealand				
European	18 (64%)	6 (60%)	24 (63%)	12 (60%)
Māori	2 (7%)	1 (10%)	3 (8%)	2 (10%)
Cook Island				
Māori	1 (4%)	1 (10%)	2 (5%)	0
Tongan	0	1 (10%)	1 (3%)	1 (5%)
Chinese	2 (7%)	0	2 (5%)	2 (10%)
Indian	1 (4%)	0	1 (3%)	3 (15%)
Other	4 (14%)	1 (10%)	5 (13%)	0

Qualitative data analysis revealed three distinct themes (see additional file 1: Interview questions and corresponding themes). Differences in responses were found between attenders and non-attenders, but no notable differences were found across sex, age or ethnicity groups. Direct quotes from participants supporting each theme are found in additional file 2: Supporting quotes.

### Theme 1: attenders found CR services reassuring and useful

Theme 1 described attenders’ perceptions of CR. Feedback on current CR services was overwhelmingly positive. CR attenders enjoyed the sessions and found them useful, partly due to the interesting and meaningful content, and because it was “easy to understand” (Attender). Attenders discussed the value of being able to ask CRNs and guest speakers (cardiologists, dietitian) questions, as it tailored the content to their specific needs and interests. One of the most highly regarded features of the CR sessions was the group environment. Attenders revealed the support from fellow attenders was comforting and they valued being able to talk to those in a similar situation.

### Theme 2: time, transport, and illness were barriers encountered by non-attenders

Theme 2 revealed the majority of non-attenders would have liked to attend CR but encountered significant barriers, such as feeling unwell or illness. Transport difficulties were a significant barrier, including a lack of access to public transport or relying on others to drive them to the sessions, which was not always possible. A few non-attenders revealed CR was not a priority because they were either not interested or were too busy.

The CRNs identified barriers to attendance which mirrored the participants’ responses. CRNs contributed additional insight in that language was another barrier as information delivered to a group in English may be difficult for some to understand. After discussing their perceptions about CR services, the researcher introduced the proposed mHealth exercise program and described how CR information would be delivered by mobile phone text messages supplemented with a website containing video messages from medical professionals and peer role models. Theme 3 revealed participants’ perspectives of the proposed program.

### Theme 3: technology can conquer barriers, but can be a barrier in itself

Attenders, non-attenders, and CRNs considered a mHealth program to be an effective way to reach people, particularly for those who could not attend CR due to a lack of time or transport, or due to ill-health.

CRNs highlighted the value and need for a mHealth program because of the high rates of non-attendance at CR sessions. In addition, it was thought that a mHealth approach could reduce existing burden on the hospital exercise CR program. The CRNs discussed how there was a lack of space for patients who would like to attend and a lack of support resources to keep patients on track once the program finished. A home-based exercise program, such as the mHealth exercise program, was viewed as a way to help some patients get started or maintain their exercise routine.

All 38 patient participants stated the mHealth program would be an effective method to deliver CR. Attenders thought the mHealth program was a good opportunity to gain extra CR, while non-attenders felt it could help them make lifestyle changes, such as starting an exercise program. Participants wanted information about what type of exercise to do, how often to exercise, and “helpful tips on little ways to get exercising” (Non-attender).

Participants provided feedback specific to the three components of mHealth: text messages, video messages, and the website. Non-attenders in particular thought the mHealth text messages would motivate them to make lifestyle changes. They felt receiving text messages would push them to do their exercise for the day, even if at times it might be a bit intrusive.

When discussing the role model video clips for the website, participants thought it would be equally important to include medical professionals and clips from patients like themselves who have gone through a similar process. Patients could then learn how people like them have made positive lifestyle changes, which would motivate them to do the same. By seeing that others have been through a similar experience, this could enhance a sense of group membership, something valued highly by CR attenders in theme 1.

A study website with CR information, video clips, and links to other trusted services and sources of information was perceived to complement the text messages. Participants felt it would be beneficial to have a source of information that explained everything in detail, step by step, that could be printed to share with others.

One of 28 attenders (4%) and 3 out of 10 (30%) of non-attenders thought that a mHealth program would be better for the younger generation and people who were frequent mobile phone users. Some participants did not have mobile phones (attenders = 4/38; non-attenders = 2/10) or access to the internet and had concerns as they were “not brought up with all that kind of stuff” (Non-attender).

While some participants considered the mHealth program might be of greater value to younger people, not all older participants were of this opinion. There were many instances where participants in their senior years were interested in taking part, but felt they would need some assistance. The 3 CRNs also considered age a potential barrier of mHealth, however with some minimal input, they stated that participants could easily be taught to read texts and access the website. Overall 3 out of 3 of the CRNs and 38 out of 38 patients (100%) considered the mHealth program would be a positive addition to existing CR services.

### Initial development of the intervention: applying focus group feedback

Results of the qualitative research were presented to the content advisory group for discussion. Based on the positive feedback the content advisory group considered it appropriate to move forward and develop the HEART intervention (Heart Exercise and Remote Technologies). Specific feedback from the qualitative research was integrated into the mHealth intervention content where possible.

The issue of non-use or infrequent use of mobile phones, particularly by the older population, resulted in additions to the study protocol. It was decided that the SMS intervention would primarily use a ‘push’ approach, so that messages would be sent to participants, requiring minimal input from them or technical ability. In addition, it was decided that researchers would teach participants how to open texts and view messages, and provide assistance to those who encountered difficulties.

Initially we planned to deliver video messages via mobile phones as we had done in a previous study with young people [21], however formative research revealed 34 out of 38 (89%) participants did not feel confident to access video messages on their mobile phone due to lack of knowledge or the cost of receiving these messages. Accordingly, the intervention was modified to allow delivery of video messages via a secure participant website.

Feedback from the formative research was used to inform the development of the intervention content. In response, a library of text and video messages was developed by investigators (LP, RM, RW, GK), using a Self-efficacy Theory framework [23] and published exercise guidelines [24] for people with CVD.

Text messages were categorized according to their exercise prescription or behavioral change focus. Self-efficacy is one’s perceived capabilities to perform a behavior in a certain situation [23] and is a key psychosocial determinant of adherence to CR [26-28]. The intervention text messages aimed to increase participants’ perceived confidence to perform exercise, overcome barriers, and schedule exercise on a daily basis, as well as increase motivation to be active.

Brief (30–60 sec) videoed vignettes of role models were developed for participants to view. Role models were identified by CRNs at various hospitals and at a CR exercise clinic. The videos were unscripted, facilitating role models to deliver their personal stories in a more natural way. Vignettes from cardiologists, cardiac rehabilitation nurse specialists, and exercise physiologists were included, which outlined the benefits of exercise, physiological responses and safety issues.

### Study 2: pre-testing study methods

From our library of messages we selected 6 text and 6 video messages to be evaluated by participants using a closed password protected online survey. The chosen messages represented the underpinnings of all messages, which was equivalent to approximately 5% of the total message library. A total of 12 messages were selected to ensure the survey took less than 30 minutes to complete, in order to retain participant interest. The online survey allowed participants to provide feedback about the content of the SMS and video messages and provided a platform to test whether participants could view the videos on their computers. A total of 52 items were asked over 10 pages using adaptive questioning and most questions were mandatory. The online survey was conducted according to the CHERRIES statement [29], a checklist designed to strengthen the quality of online survey results, and was tested by members of the research team (LP, GK, KC, RW).

Participants were a purposive sample recruited from community CR education sessions over a two month period. They had to have attended at least one session and have access to the internet. This was a different sample from the qualitative study; however two online survey participants had also participated in the qualitative study. Potential participants were sent email invites to complete the survey. Participants received information sheets outlining the purpose and length of the survey, and informed consent was completed online prior to starting the survey.

The survey used LimeSurvey® software and allowed data to be stored on secure servers at the research center. Responses were automatically captured and relevant data were extracted once the target sample size (n = 20 completed surveys) was reached. Participants who volunteered their time to complete the survey were entered into a draw to win a $100 gift certificate.

### Pre-testing study results

Participants were assigned tokens to assure anonymity and unique responses, valid for approximately one month. A total of 41 people registered their interest in participating in the survey, and 28 participated (participation rate = 68%). Of the 28 submitted surveys, only 20 were found to be complete (71%) and were included in the subsequent data analysis. Three of the 8 participants who did not complete the survey reported technical difficulties viewing the videos. Participant demographics are described in Table 1.

Survey results revealed that participants generally enjoyed the videos and found them useful. Seventeen out of 20 (85%) participants indicated they would sign up to a CR program that delivered information by video messages on the internet. All 20 participants (100%) could understand the videos and most (95%) could relate to the speakers. Three of the six video clips were perceived as just the right length, while a few participants (3/20, 15%) felt the remaining three clips were too short. Nine out of 20 (45%) participants enjoyed messages from patients like themselves the most, while the remaining preferred messages from exercise experts (30%) or medical professionals (25%), indicating that a combination of the above would satisfy most. No participants were offended by the content of the videos.

Suggestions were made to improve the video messages. Participants felt that younger CVD patients and patients of varying ethnicities should be included. A variety of role models would increase the probability that future intervention participants would be able to relate to a role model, which is an important component for increasing self-efficacy to exercise. Participants also stated they wanted to see demonstrations of different exercises, to learn how to find time to exercise and how to make exercise fun.

Feedback regarding text messages was mixed and mirrored responses from the formative research. Twelve out of 20 (60%) of participants were interested in signing up for a CR program delivered by text message. Participants (19/20, 95%) could understand the messages and appreciated that abbreviations were not used. The texts were seen as a good reminder to exercise but too many messages could be perceived as nagging. Participants appreciated that someone was ‘looking out’ for them and their health, but wanted to be sure they could trust the messages and that they were accurate. Some felt that having both texts and the internet approach with videos would be beneficial. Three out of 20 participants (15%) felt the texts would not be useful to them as they did not carry their mobile phone with them or seldom used it.

### Final development of the intervention: applying pre-testing feedback

The intervention was further developed and finalized after the formative and pre-testing research. Twenty-four weeks was chosen as the intervention length to assess long term sustainability of the intervention on exercise behavior. Participants would receive 4–6 text messages per week, using a combination of exercise prescription and behavior change texts aimed at motivating participants to complete their exercise and enhance self-efficacy using approaches such as goal setting, identifying barriers, and developing strategies to overcome barriers. A total of 118 text messages were created for the intervention.

An interactive website was created to provide a platform on which participants could view video and text messages, read information about exercising with CVD, track their exercise progress using a graphing tool, develop goals on a goal setting chart, and develop strategies to overcome obstacles. Additional videos were filmed to include more role models with different backgrounds. Interesting visuals were added to the videos, particularly around exercise tips, including demonstrations of exercises that can be done around the home. One hundred and twelve video messages were created, and 2–8 video messages per week were made available to participants.

A section was also created on the website that described who created the exercise content and how exercise targets were determined. This was done to reassure participants that their exercise prescription was safe, increasing their confidence and self-efficacy to exercise. New content was programmed to appear on the website every 3–4 days. Participants were encouraged to view the website as often as they liked, but logging on once or twice per week would be sufficient. Participants could choose whether to view their text messages on the mobile phone, on the website, or both. Please see Additional file 3 for example SMS and video messages.

Steps were taken to resolve the technical difficulties encountered by some of the online survey participants. For example, instructions written into the study protocol meant researchers could demonstrate how to log on to the study website and view the videos. The website was tested on different computer systems and Internet browser versions to ensure that content could be accessed on older computers. A help section was also created on the study website, as well as contacts for technical difficulties.

### Pilot study and randomized control trial

Pilot testing of the intervention was integrated into the full randomized controlled trial (n = 170). During the study, the first 10 study participants were closely monitored for 6-weeks to ensure they received the automated program of SMS and video messages and to resolve any technical issues that arose. In addition, the first author and two co-authors (GK and KC) received all text messages in advance of intervention participants to help identify and resolve technical issues in a timely manner. Full details of the RCT protocol have been published [19] and the trial began in mid-2011.

## Discussion

This paper outlined the process of developing a mHealth intervention prior to the conduct of a large pragmatic randomized controlled trial [19]. Each step played an important role in developing an intervention that was evidence and theory based, and relevant to the target audience.

Conceptualization involved the application of theory and evidence to improve the effectiveness of interventions [30]. In the CR setting, self-efficacy theory has been the predominant social cognitive model to be examined. Research has shown that exercise related efficacy measures can act as both a determinant and consequence of CR [31]. In terms of behavior, self-efficacy theory has been shown to effectively increase exercise initiation and maintenance [32]. Thus self-efficacy was considered an appropriate theory to underpin the content development. For the components of the exercise prescription (frequency, intensity, time, and type) and the rate of exercise program progression we chose to adopt and adapt the ACSM guidelines for SMS delivery, which was tailored to participants’ initial fitness level, age and gender. This theory and evidence based approach increases the chance of intervention success by providing achievable exercise targets for participants, thereby increasing their efficacious beliefs to exercise through performance accomplishments [32].

The formative and pre-testing steps used well established research methods to investigate if CVD patients and key stakeholders were accepting of exercise CR delivered in a mHealth format. Involvement of stakeholders and consumers throughout the content design and study design ensures interventions are engaging and relevant for end-users [33]. These studies improved the content of the intervention by incorporating feedback from the target audience and helped identify technical issues which were resolved prior to starting the RCT. The high degree of support from patients and stakeholders confirmed the need for and acceptability of a mHealth CR program, which encouraged researchers to proceed with the six month RCT. These results reflect feedback collected from mentors and patients in earlier research evaluating the acceptability of delivering a 6 week CR program by mobile phone and internet [34].

For some, the mHealth format was considered to be of particular value for younger patients who experience difficulties attending center-based CR due to transportation or time barriers. As this program is delivered directly to patients, thereby eliminating travel costs, it has the potential to benefit patients from lower socioeconomic positions. This is important because low socioeconomic status has been shown to be associated with increased CVD prevalence and mortality [35,36]. A recent (2009) review argued that in addition to improving health outcomes, mobile technology may reduce disparities by successfully reaching more deprived communities [16].

Age is often perceived as a potential barrier to mHealth, yet older participants in the present study were interested in such a program. There is a dearth of research evidence on the receptiveness of older population to mHealth programs. A systematic review examining mHealth behavior change interventions for disease management and prevention revealed no studies using mHealth for older populations [37]. The formative research in this paper suggests that older adults are willing and able to participate in such programs.

MHealth is easily customized to meet individual patient requirements. MHealth can potentially be an important component of health care reform due to the pervasiveness of mobile technology in society as well as its ability to tailor information to patients. One such model of health care delivery reform is the Patient-Centered Medical Home (PCMH) [38]. A cornerstone of PCMH is patient-centered care, where care is tailored to meet patient needs and preferences. Patients are viewed as being more active in self-management of their care, rather than passive recipients of information. A mHealth program initiated by the physician with the patient would encourage patient self-management by improving patient- physician communication, motivating patients to make lifestyle changes, and self-manage their condition.

While a mHealth CR program is promising, it may not be suitable for all patients. Some patients with co-morbidities may benefit more from greater levels of supervision offered at center-based programs, however in this sample ill-health was found to be a significant barrier to attending such programs. Exercise is the cornerstone of CR and mHealth programs offer an alternative delivery approach to increase reach and accessibility. Our current trial [19] will determine the effectiveness and safety of a mHealth delivered exercise program. However as with all CR programs, assessment of participant’s readiness to exercise is required [24].

### Limitations

The content development process was important to determine the usability and interest in a mHealth CR program. As highlighted by Whittaker et al. [20] a primary concern associated with this content development approach is the length of time required to develop the content and conduct a randomized controlled trial (2-4 years). This is sometimes seen as too long for interventions where the technology itself continues to evolve and, perhaps more importantly, how people use the technology in their daily lives changes over time. During the content development in the present study unanticipated delays occurred during video and website production.

Technical issues arose during the pre-testing study as participants were required to have broadband internet connection and the most recent version of Adobe Flash Player, preventing some patients from participating in the study. Future studies involving content development for mHealth interventions need to anticipate and plan for such issues. Testing messages via online survey can be time consuming, which is why we limited the pre-testing message library to ~5% of the total messages. Although the pre-testing library represented the underpinnings of the entire library, it was a limitation to test so few messages.

Limitations of the formative research and pre-testing studies include that the sample sizes were small and therefore not generalizable to the entire population. The studies were conducted in one large metropolitan area. Patients in rural communities may have different viewpoints about mHealth. Future studies should evaluate perspectives from a wider sample. Only 1 coder analyzed the focus group and interview data, which was a limitation, however peer debriefings were consistently performed to establish credibility [25].

Although time consuming (approximately 6 months) the detailed process was essential to ensure the potential viability of this approach.

## Conclusions

Multiple approaches to CR, such as a combination of mHealth and center-based CR, will enhance the likelihood of meeting the needs of more patients. MHealth has the potential to enhance access and delivery of CR. People with existing CVD appear to be accepting of this approach. An evidence-based, integrated, collaborative, participatory approach is needed to develop content that is likely to have the greatest chance of effectiveness.

## Abbreviations

CR, Cardiac rehabilitation; mHealth, Mobile phone and internet-based health intervention; SMS, Short message service; CRNs, Cardiac rehabilitation nurse specialists.

## Competing interests

The authors declare that they have no competing interests.

## Authors’ contributions

Principal responsibility for the study design is assumed by RM. LP conducted the formative and pre-testing research studies, analyzed the data and drafted the manuscript. LP, GK, KC, RM, RW, LD and RS were involved in content production. All authors contributed to the conceptualization stage of development. All authors read and revised drafts and approved the final manuscript.

## Pre-publication history

The pre-publication history for this paper can be accessed here:

http://www.biomedcentral.com/1471-2261/12/36/prepub

## Supplementary Material

Additional file 1 Interview questions and corresponding themes. This Microsoft word file describes the qualitative study research questions, the semistructured interview questions we asked attenders and non-attenders of cardiac rehabilitation, and the corresponding themes that emerged from the data. Click here for file

Additional file 2 Supporting quotes. This Microsoft word file lists a selection of quotes from participants in the qualitative study to support the themes discussed in the results section of the manuscript. Click here for file

Additional file 3 Example text and video messages. This Microsoft word file lists a selection of text messages developed for the intervention. Descriptions of the video messages are also included. Click here for file
