# Supplementary material for: A mHealth cardiac rehabilitation exercise intervention: findings from content development studies
Source: BMC Cardiovasc Disord. 2012 May 30;12:36. doi: 10.1186/1471-2261-12-36 (PMC3442998; doi:10.1186/1471-2261-12-36)
Supplement: Additional file 1 — Interview questions and corresponding themes. This Microsoft word file describes the qualitative study research questions, the semistructured interview questions we asked attenders and non-attenders of cardiac rehabilitation, and the corresponding themes that emerged from the data. [file 1471-2261-12-36-S1.docx]

Additional File 1. Interview questions and corresponding themes

| Research Question | Interview Questions | Corresponding Theme |
| --- | --- | --- |
| 1. What are the experiences of current ‘users’ and what are their perceptions of cardiac rehabilitation services? | When the cardiac education sessions were first explained to you what did you think of them?  For the interview with *attenders*:   1. What have you liked/disliked about the cardiac education sessions? 2. Did you find it to be a supportive environment? | Theme 1: Attenders found CR services reassuring and useful |
| 1. What are the barriers that prevent people attending cardiac rehabilitation? | For the interviews with *non-attenders*:   1. Why didn’t you attend the education sessions? 2. Did you search out any cardiac rehab information on your own? | Theme 2: Time, transport, and illness were barriers encountered by non-attenders |
| 1. What is the perceived acceptability of a proposed m-health cardiac rehabilitation program for both users and non-users of cardiac rehabilitation? How do cardiac rehabilitation nurses perceive the acceptability and usability of a potential m-health cardiac rehabilitation program? | *Mobile phone usage*   - 1. How often do you use your mobile phone?   2. What do you use it for?   3. What types of things do you feel comfortable doing on a mobile? (Calling? TXT? PXT? Videos?)   *Perceived acceptability*   - 1. What do you think about this idea?   2. What types of messages and how often?   3. Who would they want to receive messages from?   4. What appeals and why?   5. Would something like this improve any of the issues you have raised above (e.g. why they didn’t attend or what you didn’t like about it)? | Theme 3: Technology can conquer barriers, but can be a barrier in itself |
