# Supplementary material for: A mHealth cardiac rehabilitation exercise intervention: findings from content development studies
Source: BMC Cardiovasc Disord. 2012 May 30;12:36. doi: 10.1186/1471-2261-12-36 (PMC3442998; doi:10.1186/1471-2261-12-36)
Supplement: Additional file 2 — Supporting quotes. This Microsoft word file lists a selection of quotes from participants in the qualitative study to support the themes discussed in the results section of the manuscript. [file 1471-2261-12-36-S2.docx]

Additional File 2. Supporting quotes

Theme 1: Attenders found CR services reassuring and useful

*I wanted to know more information because when you come out of hospital, you have your chat with your cardiologist, next minute you’re discharged and you know a few things are being said to you, you’re going to have to watch your diet and this sort of thing, it was reassuring to know that there was going to be a follow-up with rehabilitation (Day session attender)*

*It was nice and basic. I found it easy to understand. They had you know little tips and they explained what different words mean, made it really easy (Day attender)*

*One of the problems when you’ve had a heart problem, the immediate problem’s that your brain’s not functioning quite as well as it should be…And you don’t think of things. I mean you can go along to your next cardiologist’s appointment and you’ll only ask half the things that you wanted to ask. Whereas you come along to one of these sessions, if you don’t ask it yourself someone else in the room is going to, and to have somebody that will stand there and spend the time and thoroughly explain something is just wonderful. (Day attender)*

*Others people’s experiences I think are the most, the best help that you can possibly have, because it’s scary. It’s really frightening having heart problems. And to hear what other people are going through I think makes a big difference (Day attender)*

Theme 2: Time, transport, and illness were barriers encountered by non-attenders

*I’m happy to come to these things until I get the clearance [to work] but as soon as I get the clearance I’ll go back to work (Day attender)*

*If you live in the city but you don’t have a car, you know public transport only drops you at the end of the road and it’s pouring with rain you think oh bugger it, I won’t bother you know. (Evening attender)*

*Mainly because of transport – I’ve got no transport. I’d have to catch buses and I’ve got no money for buses. (Non-attender)*

*To be honest, I was so depressed I didn’t want to leave the house. Um another part of it was um I didn’t have, I don’t drive so I didn’t have the um support of someone taking me there and bringing me back. I didn’t want to burden anybody. (Non-attender)*

*It just seemed inconvenient at the time and it wasn’t a high priority. (Non-attender)*

*A lot them, people you know like have got limited English. They’ve sort of got enough English on a one-to-one level to understand, but you put them in a group situation and they haven’t got the language skills to cope with that (CRN)*

Theme 3: Technology can conquer barriers, but can be a barrier in itself

*Well some people might not have a car to get to the place but they may have a computer with the internet so yeah. (Day attender)*

*Just for people that are isolated because a lot of people, you know, my age can’t get around, can’t get out, you know. (Non-attender)*

*Well it’s good to have other ways to reach people. Like with your multimedia, with your, different strokes for different folks. Personalize the approach. I suppose until your try it you don’t know. (CRN)*

*We don’t really have any other options. We do have…a long term exercise group who come once a week, but were at maximum capacity at the moment (CRN)*

*Because once you start getting those [texts] saying that you should be doing so many minutes a day you think, oh god I suppose I better go and do it, you know. (Non-attender)*

*[The video messages are] good I think, the blend of the practical and the professional I think is top class. Some people would align more with the professional, but others would align more with the practical and some would align with both. You know listening to this guy sort of like whatever he’s saying, talking about his own experiences, you can associate yourself with that, you know and, and a real live patient, not an actor that’s been wheeled in (Day attender)*

*I think it’s much better being online and seeing pictures and other people talking rather than just, you know, text on the phone – it’s a bit different. It doesn’t sort of sink home as much, you know. (Non-attender)*

*With our generation you don’t have computers and cell phones really. You know, we’re not brought up with all that kind of stuff…I don’t think our generations in the 60s would, um, warm to it or use it as much as a younger generation, you know. I think we’re more um hands-on sort of generation. (Non-attender)*

*Well I just think my dad, whose 78 almost 79, I’ve managed to teach him to text, and he can respond to that (CRN)*
