# Supplementary material for: A mHealth cardiac rehabilitation exercise intervention: findings from content development studies
Source: BMC Cardiovasc Disord. 2012 May 30;12:36. doi: 10.1186/1471-2261-12-36 (PMC3442998; doi:10.1186/1471-2261-12-36)
Supplement: Additional file 3 — Example text and video messages. This Microsoft word file lists a selection of text messages developed for the intervention. Descriptions of the video messages are also included. [file 1471-2261-12-36-S3.docx]

Additional File 3: Example text and video messages

Text messages

| HEART: On the website you can learn how to rate your effort when exercising. Use the scale regularly to check that you’re exercising in the safe zone |
| --- |
| HEART Rx: Your walks should be at a light intensity. You might be breathing more heavily but you should be able to continue fairly comfortably |
| HEART: When you exercise you may get short of breath, your heart rate will increase and you may sweat. This is normal and will get easier in time |
| HEART Rx: For week 2 try walking or doing some other exercise for 15min at a light pace 3 times this week. Warmup, walk, cooldown |
| HEART: Getting started is often the hardest step. The first step is to put your shoes on and stand at the front door. |
| HEART: Some people like to exercise in the morning and some in the afternoon. Pick a time that suits you and make exercise a habit |
| HEART: You are worth it! Your body is entitled to a fit and healthy life ...give it a chance. |

Video messages

| Construct | Person | Message description |
| --- | --- | --- |
| Interpreting Physiology | Cardiologist | What exercise feels like: Your heart rate increases, you feel short of breath, and sweaty. But if you feel tightness in chest, dizziness, stop and follow angina plan |
| Interpreting Physiology - Medication | Cardiologist | Medication is important, reduces risk of recurrent heart event. Like oil in a car, take it even if you’re feeling good, it has favourable effects on arteries |
| Exercising right-  Clothing; coping efficacy | Exercise physiologist | You don’t need a gym to exercise, just comfortable shoes and clothing, water bottle and a hat |
| Task self-efficacy; mastery experience | Exercise physiologist | Use your pedometer to count your steps. Helps with motivation |
| Benefits of PA; coping efficacy | Role model | Exercise gives me energy, jump out of bed and be positive you’re going to do it because you’ll feel great afterwards |
| What motivates me - modelling | Role model | Reminders of how I felt waking up in the recovery room motivates me to exercise |
| Benefits of PA; social support | Role model | Exercise lifted my depression and increased my confidence; meeting others helped as well |
| Exercise progression; mastery experience | Role model | Adding a lamppost each night to increase distance walked |
| Social support | Role model | Friends as motivators – have fun while you exercise, motivates you to go if you have to meet someone |
